# MULTILEVEL LIFESTYLE INTERVENTIONS ARE NEEDED TO IMPROVE DIABETES SELF-MANAGEMENT IN OLDER ADULTS

**DOI:** 10.1093/geroni/igad104.1934

**Published:** 2023-12-21

**Authors:** Yan Du, Lixin Song, Mitzi Gonzales, Sara Espinoza, Carlos Jaen, Bei Wu, Erin Finley

**Affiliations:** UT Health San Antonio, San Antonio, Texas, United States; University of Texas Health Science Center at San Antonio, San Antonio, Texas, United States; UT Health San Antonio, San Antonio, Texas, United States; Cedars-Sinai Medical Center, Los Angeles, California, United States; UT Health San Antonio, San Antonio, Texas, United States; New York University, New York City, New York, United States; UT Health San Antonio, San Antonio, Texas, United States

## Abstract

Type 2 diabetes (T2D) is a major public health concern in older adults. Physical activity (PA) and healthy eating (HE) are two major lifestyle intervention strategies in diabetes self-management for controlling glucose levels, preventing complications, and improving functionality and quality of life. This qualitative study aimed to explore perceived factors influencing PA and HE among predominately Latino older adults with T2D ((age=70.5±4.8; Latinos=73.3%). We conducted semi-structured interviews with 15 community dwelling older adults with T2D at the end of a 12-week pre- and post- study assessing self-monitoring on lifestyles and health outcomes. All interviews were recorded and transcribed. Two researchers analyzed the data independently using a combination of deductive and inductive thematical analysis. Codes were categorized into five themes based on the socio-ecological framework, and sub-themes were identified. The themes included five levels of factors: 1) individual (e.g., knowledge, self-efficacy, self-monitoring, perceived benefits and threats, health status,); 2) interpersonal (e.g., family, friend, health provider); 3) organizational (e.g., availability of physical activity equipment in senior apartments); 4) environment (e.g., culture norms); and 5) policy factors (e.g., health provider, retirement). The factors in one level could influence the factors in another level. Most current lifestyle interventions focus on individual level, and multilevel lifestyle interventions to promote healthy lifestyles, and improve health, independence, and quality of life in older adults with T2D are warranted. The input of additional stakeholders such as family members and community staff need to be accounted when designing multilevel intervention in older adults with T2D.

